# SURVIVAL ADVANTAGE OF APOE2

**DOI:** 10.1093/geroni/igz038.2313

**Published:** 2019-11-08

**Authors:** Sudha Seshadri

**Affiliations:** UT Health San Antonio, San Antonio, United States

## Abstract

Apolipoprotein E is a glycoprotein mediator and regulator of lipid transport and uptake. The APOE-ε4 allele has been associated with higher risk of Alzheimer’s disease and of mortality, but the effect of the less prevalent APOE-ε2 on survival remains elusive. We aggregated data of 38,537 individuals of European ancestry (mean age 65.5 years; 55.6% women) from six large population-based cohorts to determine the association of APOE-ε2, with survival in the general population. During a mean follow-up of 11.7 years, 17,021 individuals died. Compared with homozygous APOE-ε3 carriers, APOE-ε2 carriers were at lower risk of death (hazard ratio,95% confidence interval: 0.94,0.90-0.99; P=1.1*10-2), whereas APOE-ε4 carriers were at increased risk (HR 1.17,1.12-1.21; P=2.8*10-16). Risk was lowest for homozygous APOE-ε2 (HR 0.89,0.74-1.08), and highest for homozygous APOE-ε4 (HR 1.52,1.37-1.70). Results did not differ by sex. The association was unaltered after adjustment for baseline LDL or cardiovascular disease. Larger, multiethnic collaborations are ongoing.

